# Amnestic syndrome in the course of seronegative limbic encephalitis complicated by drug-resistant epilepsy: a case report

**DOI:** 10.3389/fimmu.2024.1416019

**Published:** 2024-08-29

**Authors:** Kacper Łoś, Joanna Kulikowska, Monika Chorąży, Alina Kułakowska, Napoleon Waszkiewicz, Beata Galińska-Skok

**Affiliations:** ^1^ Department of Psychiatry, Medical University of Bialystok, Bialystok, Poland; ^2^ Department of Neurology, Medical University of Bialystok, Bialystok, Poland

**Keywords:** seronegative limbic encephalitis, epilepsy, amnestic syndrome, psychotic symptoms, autoimmunology

## Abstract

We present the case of a 35-year-old female patient admitted to the hospital with symptoms of rapidly increasing disturbances of consciousness and fever for 48 hours. A lumbar puncture, bacteriological and virological examinations, and initial imaging studies did not show abnormalities. Brain magnetic resonance imaging (MRI), repeated several times, showed hyperintense confluent lesions in both temporal lobes and atrophy of both hippocampi. General examination, cerebrospinal fluid culture, the panel of antineuronal antibodies, and tumor markers remained negative on subsequent repeats. Despite several laboratory and imaging studies, the etiology of the disease could not be established, infections were excluded, and no autoantibodies were found. A diagnosis of probable limbic encephalitis, amnestic syndrome resulting from organic brain damage, and drug-resistant epilepsy was made. The patient, with limbic encephalitis complicated by drug-resistant status epilepticus, was treated with cycles of immunoglobulin and subsequent plasmapheresis. She was then transferred to the Department of Psychiatry for diagnosis and treatment of intermittent psychotic disorders. During hospitalization, the patient was observed to have multiple epileptic seizures with temporal and frontal morphology, amnestic syndrome with confabulations, and periodic psychotic disorders with the occurrence of visual hallucinations. Antiepileptic treatment was escalated by including cenobamate in increasing doses. To control the mental disorders, duloxetine, tiapride, and cognitive function exercises were introduced. There was a slight improvement in memory, a cessation of confabulations, and an emergence of the patient’s criticism of the symptoms presented. The psychotic symptoms subsided, and the number of epileptic seizures decreased. The described case portrays a unique co-occurrence of disease symptoms that are difficult to treat. It shows the therapeutic difficulties that can occur in patients with suspected autoimmune encephalitis. Furthermore, it shows the need for multispecialty care of a patient with psychotic symptoms in the course of epilepsy accompanied by amnestic syndrome.

## 1 Introduction

Encephalitis is a varied group of inflammatory diseases of the central nervous system (CNS). The estimated incidence of encephalitis in highly developed countries is approximately 5-10 per 100,000 population per year (1). The most commonly diagnosed cause of encephalitis is infectious factors, including encephalitis associated with the herpesviridae family or other viruses. The symptoms of autoimmune encephalitis (AE) can have a varied clinical phenotype, are initially uncharacteristic, and often take the form of behavioral disturbances. Autoimmune encephalitis is often a paraneoplastic syndrome, so there should be increased oncological vigilance in the diagnosis. Epileptic psychosis affects only approximately 6% of epileptic patients, the majority of whom are patients with a long history of epilepsy or who do not follow medical treatment. The most common form is postictal psychosis, which is associated with focal epilepsy, mainly temporal lobe epilepsy. It usually develops after cluster partial or generalized tonic-clonic seizures between 24 hours and a week after the onset of seizures.

Limbic encephalitis is dominated by impairments in short-term memory, attention, planning, and information processing, the severity of which depends on the duration of the disease (2). In addition to cognitive decline, there are also neuropsychiatric symptoms: aggression, apathy, or depression (3). Transient epileptic amnesia (TEA) is a syndrome characterized by short-term, recurrent memory impairment. It most often begins in middle age or older, and episodes of memory impairment may be the only or first clinical sign of temporal lobe epilepsy (TLE) (4). Moreover, bilateral damage to the temporal lobes, especially the hippocampus, often causes serious memory problems. It is worth mentioning that despite apparently similar structural damage, patients may differ in the pattern of impairment and preservation of memory functions (5).

## 2 Case description

A 35-year-old woman, with increasing disturbances of consciousness and fevers for 48 hours, was admitted to the hospital with suspected neuroinfection. The patient did not have somatic or psychiatric conditions in her medical history. Her family denied the patient’s use of psychoactive substances, trips abroad, exposure to toxic agents, risky sexual contact, and tick bites. The patient had previously led a stable family life with her husband, with whom she co-parented a 4-year-old son. Until the admission to the hospital, she had been professionally active.

The patient was observed to have a rising fever of up to 41 degrees Celsius in the hours before hospitalization, which did not respond to treatment with antipyretic drugs. Furthermore, 24 hours after the onset of the fever, a disturbance of consciousness appeared, and the patient remained allopsychically disoriented, which alarmed the family, who called an ambulance. In the hospital emergency department, the patient presented with impaired consciousness (Glasgow Coma Scale; GCS 9pts), and no focal neurological symptoms were detected, including meningeal symptoms.

## 3 Diagnostic assessment

**Figure 1:**
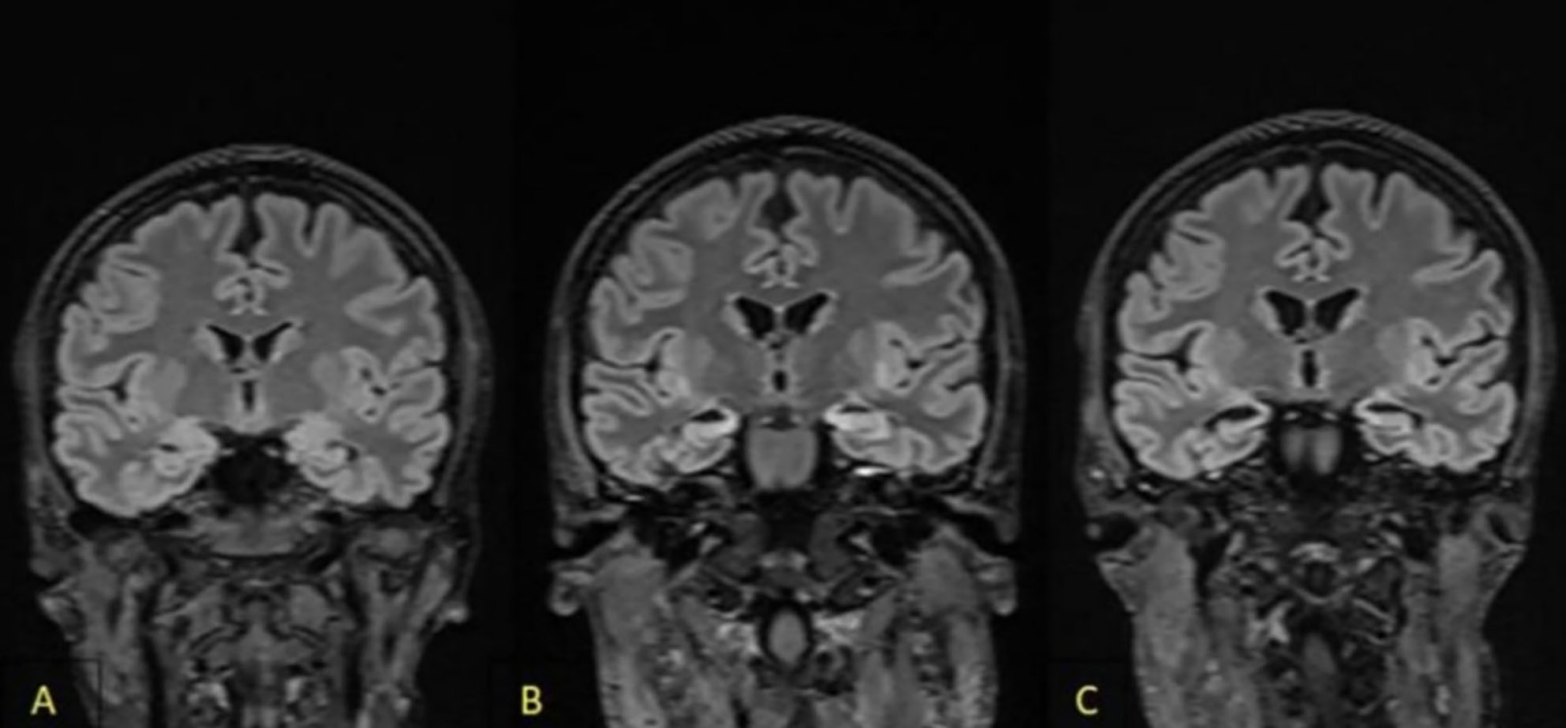
Magnetic resonance imaging (FLAIR). **(A)** On admission: hyperintensive bilateral lesions in both temporal lobes, mainly affected the hippocampi. **(B)** Hypertensive bilateral hippocampal lesions with atrophy during treatment in the intensive care unit. **(C)** Increased hippocampal atrophy during treatment in the psychiatry unit.

**Figure 2:**
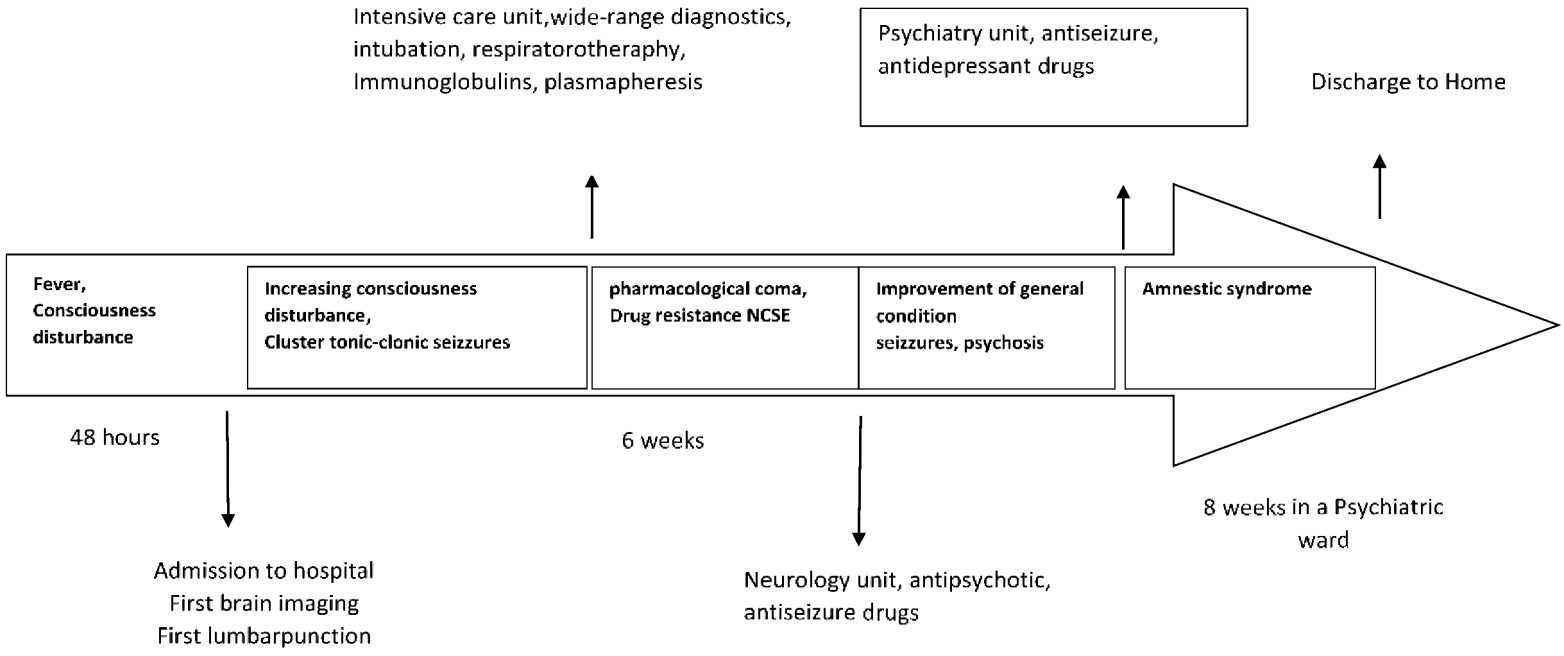
Timeline.

Computed tomography of the head was performed, which showed no abnormalities. A baseline panel of laboratory tests was conducted, which revealed no significant abnormalities. A lumbar puncture was performed and empirical treatment of meningitis was instituted, following local epidemiological guidelines. General examination of the cerebrospinal fluid also showed no abnormalities (cytosis 1 cell, total protein 44mg/dl). A cerebrospinal fluid culture was performed. On the first day of hospitalization, several generalized tonic-clonic epileptic seizures occurred. Benzodiazepines were used to interrupt the prolonged seizures, but despite the cessation of the seizures, the disturbances of consciousness increased. An electroencephalography (EEG) was performed, in which multiple, almost continuous delta waves and sharp-wave discharges were observed with predominance on the left side. A diagnosis of nonconvulsive status epilepticus (NSE) was made. A repeated lumbar puncture showed no abnormalities (cytosis 2; total protein 42mg/dl). Due to her worsening neurological condition (GCS 6pts), and lack of response to ongoing anticonvulsant treatment, the patient was intubated, mechanically ventilated, and she was put into a pharmacological coma, in which she remained for the next 46 days. Over several weeks, a brain MRI with gadolincontrast was performed several times (**Figure 1**). The result of the first examination was normal. A subsequent examination showed segmental thickening of the cerebral cortex in the left parietal-occipital lobe which was less marked in the right, without features of diffusion restriction and contrast enhancement; the description suggested that the MR image obtained might correspond to edematous changes after an epileptic seizure. A follow-up examination performed 1 week later described regression of these lesions and, after another month, described nonspecific lesions of the temporal lobes and insulae bilaterally with marked cerebral atrophy; the image suggested inflammatory lesions. Extensive diagnostic imaging of the whole body was performed and no tumor lesions were visualized. During hospitalization, a panel of neuronal autoantibodies (including surface and onconeuronalantibodies), cerebrospinal fluid cultures, a panel of tumor markers, virology tests, a rheumatology panel, and diagnostics for porphyria were performed several times; the results remained negative (1). Positive results for 14-3-3 protein and tau protein were obtained and Creutzfeldt-Jackob disease was suspected. Diagnostics were further enhanced with RT-QuIC testing, which did not detect abnormal prion which is a specific indicator of Creutzfeldt-Jakob disease (**Table 1**). During ventilator therapy and pharmacological coma, paroxysmal activity persisted in the EEG in the form of rhythmic, generalized sharp wave complexes, intensified by photostimulation, against an isoelectric line background. Taking into account the overall clinical picture, autoimmune encephalitis was suspected. According to the references from Lancet Neurology from 2016, the patient met the criteria for possible autoimmune encephalitis: subacute onset of symptoms (<3 months), presence of psychiatric symptoms, epileptic seizures that cannot be explained by another cause, and MRI features suggesting encephalitis. Although the pleocytosis was not detected in repeated analyses of the cerebrospinal fluid, according to the criteria, it was not necessary to make the diagnosis. Moreover, analyzing the guidelines, it is also possible to make a diagnosis of limbic encephalitis, which includes subacute onset with memory deficit (<3 months), bilateral MRI abnormalities in the medial parts of the temporal lobe, and epileptiform activity on EEG involving the temporal regions. Considering the probable autoimmune encephalitis and after excluding other possible causes of the patient’s symptoms, it was decided to treat her with plasmapheresis and immunoglobulins. The therapy included a supply of immunoglobulins at a dose of 2g/kg body weight and plasmapheresis. Several attempts were made to bring the patient out of her pharmacological coma, but numerous seizures and persistent disorders of consciousness were observed. After obtaining an image in the EEG with a burst-suppression pattern, it was decided to make another attempt to bring the patient out of her pharmacological coma. A gradual improvement in the patient’s general condition was noticed so the patient was transferred to the Neurology Department. In the following days, epileptic seizures of focal morphology from the temporal and frontal lobe with impaired consciousness, sometimes undergoing secondary generalization, were observed with a frequency of 3-4 times a day. Antiepileptic therapy (valproinianacid+lamotrigine+lacosamide; VPA+LTG+LCM) was escalated. Mental disturbances in the form of periodic agitation, and anxiety with accompanying psychotic symptoms such as visual hallucinations appeared. The patient refused to take medication. Due to the inability to control the mental disorder, the patient was transferred to the Department of Psychiatry to stabilize her mental state. During the psychiatric hospitalization, the patient was observed to have amnestic syndrome with confabulations and periodic disturbances of consciousness with the occurrence of visual hallucinations. The patient in the initial period of her stay in the psychiatric ward remained auto- and allopsychically confused, she declared that she came to the hospital this morning. She did not know the reasons for her admission to the ward. She did not want to take medication due to severe anxiety and had a lack of understanding of why she should take pharmacotherapy. A similar situation occurred every day for 14 days. The patient did not know that she was married and during visits she did not recognize her life partner. Periods of anxiety occurred mainly in the morning, after waking up, while in the evenings the psychotic disorders intensified. The patient manifested psychomotor restlessness, became verbally aggressive, and visually hallucinated. Due to the behavioral disorders, treatment with tiapridal was initiated; previous treatment with risperidone and haloperidol in the Neurology Department was ineffective. States of psychomotor agitation were managed with lorazepam 2.5 mg administered *ad hoc*. Initially, she required an injectable preparation due to severe agitation. Antidepressant and anti-anxiety treatment with a duloxetine preparation of up to 60mg/d was used. In the initial period, the patient’s statements were covered with numerous confabulations, an in-depth interview suggested memory gaps going back approximately 10 years. She could not remember her child, her husband, the death of her father, or any significant life situations that had occurred over a decade. Short-term memory impairment was also observed. The memory scratch reached about 2 hours, and around lunchtime, she was unable to recall what she had eaten for breakfast. During the day, her level of tension, and anxiety decreased. Assessment of cognitive function, using the Montreal Cognitive Assessment Scale (MoCA), indicated impaired short-term (immediate) and long-term memory, a significant limitation in learning new material, serial and retrograde amnesia, impaired temporal orientation, and confabulations. Immediate recall, attentional processes, perception, procedural memory, and self-psychic orientation remained undisturbed. The patient scored 19/30 in the MoCA scale. In the further stage of hospitalization, confabulations decreased in intensity, memory concerning important life events improved, and she recognized her husband. The patient was able to recognize friends, and remembered the name of the physical therapist and the attending physician, but did not recognize the faces of people she met during hospitalization (prosopagnosia). In a follow-up MoCA test, the patient scored 1 point higher for attention. She was still confused about time. The patient understood all instructions, was able to follow them correctly, and was able to perform psychological tests. Due to the persistence of epileptic seizures several times a day, cenobamate was included in her therapy at an increasing dose as recommended, achieving a significant reduction in seizure frequency at a dose of 100 mg/d. The patient was transferred to a reference epileptology department for several days of observation, where a long-term EEG study was performed and further therapeutic recommendations were made. Long-term video-EEG showed focal lesions, with a tendency to generalize, in both hemispheres of the brain with a predominance on the left side, and the clinical picture suggested seizures with temporal and frontal morphology. As a result of a prolonged stay in the intensive care unit, the patient was a non-walker with features of polyneuropathy. Motor rehabilitation was carried out in the hospital wards. Before discharge from the hospital, the patient was able to walk independently with the help of another person (**Figure 2**).

**Table 1:** Diagnostic tests performed.

Imaging examinations		
Brain MRI	Attached	
**Computed Tomography (CT) of the head** **Angio-CT of the head and neck** **CT of the chest with contrast** **CT of the abdomen and pelvis with contrast,** **Transvaginal ultrasound** **Ultrasound of the thyroid gland** **Cystoscopy** **Colonoscopy** **Gastroscopy** **Positron emission tomography of the whole body**	**Without any clinically significant abnormalities**	
The most important laboratory tests		
Cerebrospinal fluid (CSF)culture		Negative
**Bacteriological tests (RT-PCR);** H. Influenza, N. meningitidis, S. Pneumonia, Streptococcus gr. B (S. Agalactiae), L. Monosytogenes, E.coli K1,		**Negative**
**Serological tests** IgG and IgM antibodies against Borrelia burgdorferi (CSF+ serum) IgG and IgM against Toxoplasma gondii Tick-borne encephalitis (IgG+IgM: CSF + serum) Anti-HIV Anti-HCV Anti-HBs VDRL (CSF+ serum)		
**Virological tests (RT-PCR; CSF + plasma)** CMV, EBV, HSV1,HSV2,HHV-6,HHV-7, VZV, MV, B19V, AdV, HEV, HPeV, SARS-CoV-2		**Negative**
**Surface antigens (CSF + serum)** NMDA, AMPA1, AMPA2, DPPX, LGI1, CASPR2, GABAB		**Negative**
**Onconeuronal antibodies:** **Hu, Yo, Ri, Ma2/Ta, CV2, amphiphisin, recoverin, SOX1, titin, Zic4, GAD65 i Tr (DNER)**		**Negative**
**Isoelectrofocusing of the cerebrospinal fluid**		**Oligoclonal stration type 4**
Evaluation of total complement activity, p-ANCA, c-ANCA, ANA; antibodies to phospholipids, ANA Plus profile (JO-1, PL-7, ZOB, profile anti-MPO, neuronal IgG profile; antibodies to beta2glycoprotein, cyclic citrulline, granulocytic cytoplasm, cardiolipins, myeloperoxidase, prothrombin, phospholipase receptors		**Negative**
**Tau protein (Erlangen Score (ES))**		**High tau protein values >362 pg/mL**
**14-3-3 protein**		**Positive**
**RT-QuIC**		**Negative**
Tests for porphyria - Determination of excretion of porphyrin precursors (delta-aminolevulinic acid (ALA) and porphobilinogen (PBG)) and porphyrins in urine - Determination of the fluorescence spectrum of porphyrins in plasma		**Negative**

## 4 Discussion

The presented case describes a patient with limbic seronegative encephalitis complicated by amnestic syndrome and drug-resistant epilepsy. Despite a wide panel of studies performed, it was not possible to find an etiology of the disease. It is also very difficult to choose the appropriate pharmacological treatment for a patient with the presented symptoms, especially because of the potential side effects of drugs. In most cases of encephalitis, pleocytosis is observed in the cerebrospinal fluid; a study by J. Héberte et al. on a cohort of 95 patients with AE showed that in approximately 30% of cases, the cerebrospinal fluid remained normal (6). The most common cause of encephalitis is viral etiology; however, subsequent repeat PCR tests from both her blood and CSF for viruses have remained negative (1). The dynamics of the changes in the subsequent repeated MRI examinations indicated bilateral involvement of the temporal lobes, mainly the medial area (hippocampus) in the form of hyperintense lesions mainly visible in T2 and FLAIR sequences. Analyzing the diagnostic criteria for AE, it was found that the patient met the diagnostic criteria for probable autoimmune encephalitis: subacute onset, disturbance of consciousness, presence of short-term memory disorders, seizures that could not be explained by any other cause, and MRI signs suggestive of encephalitis. At the same time, the patient met the diagnostic criteria for limbic encephalitis: subacute onset, seizures, memory disturbances, psychiatric symptoms suggestive of temporal lobe involvement, bilateral MRI lesions involving the temporal lobe, and an epileptogenic EEG pattern involving the temporal region. It should be emphasized that although the presence of autoantibodies is not necessary for the diagnosis of limbic encephalitis, they should be actively sought because of possible clinical implications. In the described patient, autoantibodies could not be identified, but despite this, an extensive oncological screening was performed, in which no proliferative lesions were found. It is worth noting that such a group of patients should remain under the close care of physicians, who should demonstrate high oncological vigilance. The treatment included cycles of immunoglobulin and plasmapheresis, after which the patient was successfully brought out of a drug coma. Improvement of her clinical condition after immunotherapy indicated a likely ongoing autoimmune process. It should be noted that the diagnosis of autoimmune encephalitis is a difficult one to make, and even the detection of autoantibodies does not make the diagnosis certain. A study conducted by E.P. Flanagan et al. from the Mayo Clinic shows that approximately 1/3 of autoimmune encephalitis diagnoses are incorrect, and the most common reason is the incorrect interpretation of the detected autoantibodies (7). Infections are a potential factor in the immune response. Infectious agents (bacteria, viruses, parasites) can lead to the development of autoimmune disease through molecular mimicry. According to this theory, infection with a virus or bacteria with epitopes that show similarity to host antigens can lead to the activation of autoreactive lymphocytes and the development of a response to their antigens, the expression of which increases under the influence of fever, local hypoxia, and increased production of reactive oxygen species accompanying the infection. The production of autoantibodies, as a consequence, can lead to symptoms characteristic of autoimmune encephalitis. In the described patient, the agent that could have caused the infection was not found. It should be noted that there may be mutations of various pathogens that we have not yet detected, which may not show up in potential tests (8). In the course of the investigation, a positive result for the 14-3-3 protein was obtained, so the suspicion of Creutzfeldt-Jakob disease was raised; however, significant clinical improvement after treatment ruled out this disease entity. It is worth remembering that the 14-3-3 protein is a non-specific protein, has high sensitivity but low specificity, and is found in many diseases with neurodegeneration.

For many years, there have been reports about the important role of the gut microbiome and the gut-brain axis in the pathogenesis of neurological and psychiatric diseases (9). The mechanism is not clearly understood, however, on the one hand, the initiation of neurodegeneration in the gastrointestinal tract is emphasized, and on the other hand, the activation of inflammatory processes that are reflected in autoimmune processes, for example, in animal models of multiple sclerosis (10, 11). Many authors raise the inflammatory hypothesis as a model to explain the development of psychiatric diseases such as depression. In the described patient, the gut microbiota was not studied, however, the possibility should be noted of the co-occurrence of multiple biological mechanisms and molecular pathways leading to encephalitis without an identified etiologic factor. Perhaps in the future, in similar patients, procedures in the form of intestinal microbiota transplantation or dietary interventions or not yet described procedures will be one of the elements of therapy or prevention of relapse (12).

Since the beginning of the pandemic, questions have been raised about the impact of the SARS-CoV-2 virus on the nervous system (13). On the one hand, a link to neurodegenerative processes is suggested, and on the other hand to inflammatory processes. The molecular mimicry mechanism mentioned earlier as well as hyperstimulation of the immune system may also play a role. The initiation of autoimmune processes is also discussed as a possible complication after vaccination against SARS-CoV-2, mainly with a mechanism of mRNA (14). The described patient was vaccinated with the mRNA vaccine (three doses, the last one 12 months before the onset of the disease), while current diagnostic capabilities, as well as available literature data, do not currently allow verification of such a hypothesis. This underscores the role of publishing as many clinical cases of unclear etiology as possible from different regions of the world, which perhaps in some time will enable their joint analysis and verification of certain hypotheses.

Very numerous (several times a day) epileptic seizures with temporal and frontal morphology were observed. The applied and modified pharmacotherapy (VPA+LTG+LCM) did not bring clinically significant improvement. Only the use of cenobamate (from a dose of 100mg/d), the efficacy of which has been confirmed in drug-resistant epilepsies, resulted in a reduction in seizure frequency (15). Psychiatric symptoms, including psychosis, are one of the most common and often observed symptoms in autoimmune and limbic encephalitis (60% of cases) (16). In the described patient, psychiatric symptoms in the form of psychotic symptoms with concurrent visual hallucinations appeared after several months of illness. It was noted that they were probably related to concurrent and difficult-to-control epileptic seizures of focal onset. Due to the duration of epileptic seizures in correlation with psychosis, it was not possible to diagnose post-epileptic psychosis (according to the definition of >24h) or interictal psychosis, which most often occurs in patients with a long duration of illness (>15 years) (17). The occurrence of psychosis is in a significant relationship with the frequency of epileptic seizures. The temporal morphology on peri-ictal psychosis is often difficult to distinguish from seizures with impairedconsciousness. (18). It is worth noting the damage to the patient’s CNS, including the hippocampus, and her characteristics involving severe anxiety. Benzodiazepines and low-dose antipsychotics are used in the treatment of epileptic psychoses, but guidelines for the treatment of this group of patients have not been established thus far. A significant complication of most neuroleptics is the lowering of the seizure threshold, which, with the occurrence of 4-6 seizures per day in the described patient, was a particular clinical challenge. Another significant difficulty was the selection of a neuroleptic that would be effective in controlling psychotic disorders, while at the same time not causing the deterioration of cognitive functions present in a patient with amnesia. Initially, in the Neurology Department, psychotic symptoms were treated with haloperidol to 5mg/d and then with risperidone up to 4mg/d, without clinical success. After transfer to the psychiatry department, due to the ineffectiveness of previous treatment and the patient’s significant risk of lowering her seizure threshold, it was decided to treat with tiapride up to 100mg/d. Tiapride is the drug that has the least effect among neuroleptics on lowering the seizure threshold (19). Cognitive impairment is also a significant side effect of neuroleptic treatment. Given the small effect of tiapride on the ability to remember new information and the small sedative effect, it was decided to prescribe this drug (20). The antidepressant and anti-anxiety drug duloxetine was included, which, in addition, by increasing the secretion of interleukin 10 (IL-10), has the potential to reduce the body’s inflammatory process (21). In addition, it is an anti-anxiety drug with properties that improve cognitive function. A significant symptom resulting from organic brain damage in the described patient was amnestic syndrome. It is known that non-progressive bilateral temporal lobe damage in humans causes severe amnesia (5). In particular, they have been identified as playing a key role as amnesia has been reported in patients with bilateral lesions confined to this structure (hippocampal amnesia) (22). Typically, patients with hippocampal amnesia experience significant problems in acquiring new episodic memories, such as personally experienced autobiographical events. This atherotroic amnesia is widely credited with typifying such patients. However, much controversy remains about how to characterize the aspects of memory loss associated with bilateral hippocampal lesions (23). During the neuropsychological examination, the patient understood the questions, correctly acquired and extracted new information, and tried to perform various operations in direct memory, but there were significant difficulties in maintaining attention. She created statements appropriate to the questions asked, experienced profound disorder in remembering new information, and had retrograde amnesia with a temporal gradient. At the time of the examination, the patient was aware of the memory deficit. There were disturbances in encoding information into long-term memory. The disease picture suggested an amnesic syndrome associated with damage to the medial temporal lobes. During her hospital stay, psychoeducation was also applied, cognitive functions were exercised, and there was only a 1-point improvement in the MoCa test. The patient consolidated her memory regarding important life events, remembered the names of her husband and child, and recognized their faces. However, she was not able to remember new faces and she only learned 2 new names: those of the attending physician and the rehabilitation therapist. Prosopagnosia may be related to damage to temporal lobe structures, which was visualized in the brain MR examination of the described patient. In the further course of treatment, the patient was cooperative and satisfied with the prospect of being discharged from the hospital and returning to her family.

The presented case describes an extremely rare co-occurrence of difficult-to-treat disease symptoms. The case draws attention to the therapeutic difficulties that can occur in patients with suspected autoimmune encephalitis and demonstrates the need for multispecialty care of patients with psychotic symptoms in the course of epilepsy accompanied by amnestic syndrome. Finally, the case demonstrates that we are still not always able to determine the etiology of the disease.

## Data Availability

The original contributions presented in the study are included in the article/supplementary material. Further inquiries can be directed to the corresponding author.
